# Reports on boys’, youth’s and men’s health in Canadian newspapers: Now what?

**DOI:** 10.15171/hpp.2017.27

**Published:** 2017-06-14

**Authors:** Margareth Santos Zanchetta, Aaron Andrew Byam, Donna Solomon, Katayoon Jalili, Carlos Haag, Silvia Tallarico

**Affiliations:** ^1^Ryerson University, Toronto, ON, Canada; ^2^St. Joseph’s Health Centre, Toronto, ON, Canada; ^3^Hannan Fertility Centre, Toronto, ON, Canada; ^4^Toronto Western Hospital, University Health Network, Toronto, ON, Canada; ^5^Independent researcher, Toronto, ON, Canada; ^6^Canadian Association for Equity, Toronto, ON, Canada

**Keywords:** Men’s health, Mass media, Health promotion

## Abstract

**Background: ** This media content analysis explored the Canadian newspapers reporting on men's health, and their contribution to public understanding of the social determinants of men’s health and lifestyles.

**Methods: ** A media content analysis of 44 news articles on boys’, youth’s and men’s health,published from 2010 to 2014 by three national newspapers (The Globe and Mail, National Post,and Metro News).

**Results:** Data indicated that the predominant discourse consists of informative and awareness messages, mostly about men’s prostate and sexual health. Very little health news content referred to working conditions, education and income, all of which are significant social determinants of health (SDH). This may reflect the current state of health research, which does not adequately incorporate the effects of these determinants. It may also indicate a reproduction of dominant health knowledge and understanding of masculinity. Little content was found on policy solutions to other publicized health issues, such as limited access to health services or inter-sectoral collaborations; this reflects a lack of government action and a lack of citizen engagement toward the creation of a concerted men’s health policy.

**Conclusion:** Despite the acknowledged importance of the media in promoting access to health information and indirectly contributing to improve the general public’s level of health literacy, it is also necessary to remember that there must be a greater attention to the structural constraints imposed by socioeconomic inequalities. Future studies should explore media discourses about men’s unequal access to health care services and citizens’ awareness of ways to overcome those inequalities shortcomings.

## Introduction

In a society as multicultural as Canada, it is challenging to say how collectively health is perceived, or more specifically, in the case of this study, men’s health. The socio-cultural matrix based on oppressive patriarchy,^1^ paradoxically, entails serious damage for men too, reflected in negative health consequences. Since the cultural construction of men’s bodies remains focused on sexuality and its issues,^2^ the health effects of patriarchy on men is somewhat neglected. Men are believed to follow unhealthy lifestyles, and pay less attention to the prevention of acute and chronic diseases, presumably from a sense of masculine ‘toughness’.^3^ Therefore, issues of vulnerability are overlooked, which in turn may influence how local and national governments determine men’s health policies.

The need for a suitable approach to men’s health is gradually being recognized worldwide and promoted by various countries. The emerging alliance among the private sector, community organizations, and the general public corroborates the existence of links between media portrayals of men, men’s targeting, social policies and programming. Men’s health policies have been pioneered by Ireland,^4^ Brazil,^5^ and Australia.^6^ The Canadian Institutes of Health Research (CIHR) has recognized the need for men’s health research.^7^ The Institute of Gender and Health (IGH)^8^ within the CIHR is ensuring that research on boys, youth and men will be done to address specific health challenges.^9^ For this study, it was adopted the general denomination “boys, youth and men” as understood by CIHR.

The CIHR research framework for boys’, youth and men’s health is only a starting point, albeit an appropriate one, for scientific knowledge production in this area. More is expected to occur if the public is adequately informed and supports the idea of the development of a national men’s health policy in Canada. Within this context, the media can play a useful role by collaborating with the field of health promotion by developing and promoting an accessible discourse, promoting awareness and educating the public, raising issues to prominence, and influencing collective action. By studying newspapers, we can uncover the less evident features of men’s health promotion for the creation of supportive environments to optimize the key social determinants of men’s health. With this media role in mind, this study was designed to assess and analyze what kind of messages regarding men’s health are prominent in Canadian newspapers, a specific mass-reach medium.

### Realities and challenges in men’s health promotion 

Worldwide men’s health promotion initiatives tackle contextual, political, and philosophical issues as a matter of gender equity, involving differences in needs, power, resources and access to them, plus obstacles and opportunities.^10^ In this paper, men’s health is conceived within a framework of multiple aspects—psychological, physiological, social, cultural and environmental—that addresses the nature of boys’, youths’, and men’s health and wellness.

Canadian epidemiological statistics on the health of boys, male youth, and men reveal major health vulnerabilities also in the way men deal with risks and promote their wellness.^11^ Men have higher rates of cancer, heart disease and diabetes than women. Men have higher rates of death due to suicide, and to “violence, accident and other injuries” as compared to women; in fact, the combined category of “violence, accidents and other injuries” is the third leading cause of death in men, and the leading cause of death for male children, aged 1 to 19 years.^12^ For men living with multiple chronic diseases, statistics reveal the top health conditions are cancer, hypertension, obstructive pulmonary disease and asthma.^13-17^ Drug use is considerably more common and problematic among male youth and adult men than females, including alcohol, cannabis, ecstasy, heroine, and crack-cocaine^18^ added to high incidences of sexually transmissible diseases, such as chlamydia, gonorrhea, and infectious syphilis.^19^Modifiable risks are among the top five leading causes of death for men in Canada. A potential explanation for these findings is that Canadian society has conditioned boys and men to not prioritize health in their lives.^20^

While researchers have begun to outline gender differences and the effects of dominant masculinity in terms of the health consequences for boys, youth and men, it is needed to better understand the views and actions of men (and boys and male youth and parents) in order to shift them in more healthful directions. To reframe the understanding of what men’s health entails, it is critical to incorporate the perspective of the social determinants of health (SDH), which includes the dynamics of sex/gender. Such an approach should be understood by health care professionals, educators, and policymakers, communication professionals and the general public. This paper uncovers cues to re-posit the collective understanding of men’s health promotion issues.

This study had three objectives: (*a*) identify recurrent ideas in newspaper articles on the health of boys, youth and men in Canada as the prominent disclosure of social determinants of men’s health; (*b*) identify newspapers’ contribution to promoting general awareness of issues and challenges regarding this topic; and (*c*) discuss the potential impacts on the general public’s interest in participating in the movement for boys, youth, and men’s health. The main research question was: What types of messages about boys’, youth’s and men’s health are popular Canadian newspapers disseminating to the general public?

### Conceptual framework

The Population Health Promotion Model (PHPM)^21^ includes among the dimensions of health promotion, the SDH (e.g. social support networks, personal health practices, coping skills, access to health services), which are related to places and conditions where individuals are born, live, work and develop.^22^ Strengthening community action, creating supportive environments, developing personal skills, building a healthy public and reorienting health systems are comprehensive health promotion actions to optimize the SDH. The model also indicates levels of interventions to promote health at the individual, family and society levels. Its appropriateness relies on its capability to support an understanding of how news dissemination helps to address an initial recognition of men’s health disparities as experienced in Canada (e.g., differences in social exclusion, suicide, crime involvement and imprisonment); in addition to unhealthy expressions of masculinity.

## Materials and Methods

A media content analysis method^23,24^ to explore ideas or recurrent themes associated with the research topic in newspapers’ articles. This method proposes that a general, simple analytical question should guide the retrieval of contents to detect what is collectively acceptable and recognized in a given social group. The method explores the general orientation of the discourse (+, - or neutral), the period of document production, who produces the documents, types of subjects, the social norms and behavioural codes, the public awareness about a given subject, as well as the social interactions among individuals. Each newspaper article was considered an analytical unit.

Relevant newspaper articles were retrieved for three years according to the CIHR-IGH call for research proposals, issued in winter 2014. The call presented five CIHR-IGH research priority areas. The chosen topic was healthy lifestyle and health promotion and communication for boys and men.^25^ The choice was made based on the rationale of being it the most appealing to the general public.

The research team met bi-weekly to discuss the development of the retrieval, reading and analytical work over the course of eight months. As a strategy to establish rigor and credibility, we used an internal verification of the news reading interpretation (conducted by DS and KJ) follows. One of the male co-investigators (AAB) acted as a “natural expert” by reading extracts and reviewing their interpretation ensure there was no gender bias effect on the final interpretation of the readings. The news readers (DS and KJ) had a high degree of agreement on interpretation of the data, partly due to their shared knowledge of the SDH, acquired as former undergraduate nursing students. Despite being neophytes in the matter of men’s health, they were able to easily identify the SDH in relation to health promotion, social marketing and the political dimensions of men’s health promotion. The data analysis, within the scope of men’s health and masculinities, was led by MZ and ST, both researchers in these areas.

### Source of documents

Articles were drawn from three national newspapers as the main source of documents to be analyzed; they are easily accessed by the general public, not limited geographically and typically written in plain language. The target newspapers were: National Post, The Globe and Mail, and, Metro News. The National Post and The Globe and Mail are leading Canadian English-language national newspapers; the latter 2.4 million readers weekly.^26^ Metro News is Canada’s most read national daily newspaper and the first national newspaper to publish in both English and French; it is free, and has 1.6 million print readers daily, and 1.1 million web-visitors monthly.^27^ Articles were from both printed and online sources, and both paid and free distribution newspapers, because of their broad availability and distribution to readers of all sex, gender, and socio-economic groups, as well as reading and literacy levels. The intention was to identify which type of health news reaches a broad cross-section of the male population of readers of all ages and social conditions.

### Search strategy 

Using Google search engine, DS and KJ retrieved relevant news articles from the newspapers’ websites archives, using the key terms: ‘boys’ health’, ‘youth’s health’, and ‘men’s health’, combined with “lifestyle”. “Lifestyle” is a target area of the CIHR program. This methodological decision is justified based on the non-existence of keyword classifications in the archives, as there is in the scholarly database. The PHPM^21^ did not determine the definition of search terms. The timeline was limited to the years 2010 through 2014 because this period was the most active time for reporting related to the CIHR-IGH launch of the Boys’ and Men’s Health Program. The Program was initiated circa June 2009, and the official Program was launched in November 2013.^25^ Document retrieval ended in February 2014. A total of 56 articles were downloaded; after preliminary reading, 12 were excluded because their content was not relevant to the research objectives. Therefore, 44 articles composed the final *corpus* to be analyzed. Neither the length of articles nor their appearance throughout the newspapers nor the readers’ profile were features of interest in the analysis conducted.

### Document analysis

The analysis started with a critical reading of the 44 articles. DS and KJ identified which content was related to men’s health promotion and completed a content retrieval chart displaying recurrent themes as proposed by the analytical method.^23,24^ The chart comprised 7 columns: one for article identification (newspaper company, and date), and the remaining columns for the entry of the interpretative summaries that exemplified the 5 different message goals (elicit prevention, provide information, provoke awareness, introduce politics and unclear message), as well as a short sentence as a key, synthesis message contained in each article. Next, the content was assembled and classified by major topics (see Table 1) and message goal (see Table 2).

Message goal was established by readers who answered the following direct guide question: “What is the key idea in this sentence?” No other criteria of selection was applied, but an agreement among readers was sought. Major statements were identified and correlated with SDH (see Table 3). Finally, interpretation of key messages pointed to indicators for future major comprehensive action strategies to promote men’s health, grounded in the empirical evidence as reported by newspapers (see Table 4). The PHPM^21^ was instrumental to the analytical work in the phase of identifying SDH in each message and guided the classification of the messages’ core statements. Key media messages were then linked to the comprehensive actions for health promotion.

## Results

The results are presented in the upcoming sections according to the study’s objectives within the health promotion perspective. Results addressed issues of provision of health promotion to awaken individual’s awareness, education of preventative behaviours, as well as understanding political issues related to men’s health. The analysis was summarized within this perspective (see Table 2). Half of the retrieved messages involved the SDH. Core statements of those 24 messages and link them to 8 corresponding SDH are presented in Table 3. The analysis indicates that the core statements fit within the conceptual scope of the associated SDH. The PHPM assisted in classifying the top 10 messages as comprehensive strategies for health promotion. Some verbatim are presented as extracted from the newspapers.

### Newspapers’ topics about boys’, youth’s and men’s health 

The main health topics were arranged into Table 1 to illustrate their comparative frequencies and distribution by newspaper source. Metro News accounted for 60% of the total messages about men’s health. This is a significant result since this free newspaper likely due to its effective circulation reaches a broad readership, especially with its distribution in public transit stations, convenience stores, and other street level access points. The most common topic was prostate health and men’s sexual health; this reflects and perpetuates the stereotype that men’s health is centered on sexuality and the prostate, but also reminds the public of health risks, as the following quotes show.

“Men should be aware of their prostate cancer risk, especially those with a father who has had the disease. It almost doubles the risks…”

Mental health issues, social environment, and seeking medical attention were the lowest represented men’s health messages. This is unfortunate given the range of men’s health issues, including the rising rate of reported mood disorders from 2007 to 2011.^28^

### Messages’ goal about boys’, youth’s and men’s health

The messages were also analyzed with regards to their goal. Some articles involved multiple or mixed goals, which totaled 115 entries for 44 articles. Providing information was the most frequent goal (37.4%). Especially in relation to the SDH, this type of information pointed to associations with health outcomes, but did not suggest or promote advocacy or any political health movement and action. The following quote is an example:

“…most researchers would conclude that without comprehensive community development – housing, healthcare and education – there will never be a permanent decrease in violent crime.”

These messages might provoke certain interested readers, especially those who possess the resources to do so, to follow-up and pursue further information, and perhaps even call for action and advocacy in matters of men’s health. However, such messages offered nothing that would assist the reader in obtaining practical or instrumental information about the health topic presented, as the following quote shows:

“…one in six men will develop prostate cancer in their lifetimes…. It’s as big an issue as breast cancer… but there’s a significant gap in the level of funding and awareness.”

Despite no to do so, a culture of neutral message is expected to be changed due to the increasing seriousness of treated subjects. The least often recurring message goal introduced a political perspective or addressed government policy (7%). This type of content typically announced changes in government policy or the status of legislation and interventions intended to benefit boys’, youth’s and men’s health. This indicator of political messages suggests the need for more reflection in societal dialogue about the development of broad men’s health policies.

### Importance of the newspapers’ messages

Only 24 messages included content related to the SDH and their role in the health of boys, youth, and men. Table 3 shows that personal health practices (33%) and social support networks (17%) were the most frequently mentioned SDH. The following quotes relate men’s health to the criminal justice system issues as well as domestic violence.

“The man who created Canada’s only shelter dedicated solely to male victims of domestic violence died on Friday.… His death has sparked outrage and sadness among those who are pushing for greater recognition of men’s issues in law and family court.”

Newspapers tend to focus on certain lifestyles, health practices and attitudes that boys, youth and men exhibit; these may also mislead health professionals (see Table 3).

These results suggest that relying only on men’s disclosure of traditional symptoms could lead to an under-diagnosis of depression in men and that clinicians should consider other clues when assessing depression in men.

Some men’s misconceptions about their relationship to women were revealed:

“There seems to be this general philosophy amongst men in that age group that they can do whatever they like to women”, she said. “We need to target men and boys and say to them you are responsible for the abuse and you need to stop this behavior”

The media illustrate how boys, youth, and men are deprived of knowledge about enhancement of their personal health practices and help-seeking behaviours.

“Men’s Health Network…points at men’s upbringing as one of the reasons guys might be hesitant to see their MD…. Shockingly, they also found that more than half of premature deaths among men are preventable.”

News articles show that lifestyle and health practices require behavioural change to improve well-being. The deleterious consequences of having ineffective coping skills were reported, particularly among socially vulnerable groups:

“We found that more First Nations children presented to emergency departments for disorders secondary to substance abuse and intentional self-harm than other children.”

Very little health news content referred to working conditions, education and income, significant SDH. This may reflect the current state of health research, which does not adequately incorporate the effects of these SDH.^11^ It may also indicate a reproduction of dominant health knowledge and understanding of masculinity.

### Key messages for future health promotion actions

The key informative messages present in the news reports are promising for future health promotion actions. Table 4 summarizes the top ten messages and relates them to two major comprehensive action strategies as proposed by PHPM. Seven of the 10 messages are related to developing personal skills, frequently linking men’s health to traditional views of masculinity by portraying health as a personal responsibility as opposed to a health promotion matter within a macro societal level perspective.

“From an early age, boys are taught not to cry or complain, and being ill is seen as a weakness.”

Conversely, the key messages can also bring awareness to their scope for autonomous choice:

“I can’t oppose legislation against this because I think it’s a travesty so many kids are being harmed on a regular basis with the complicity of the medical establishment.… [If] he wants to be circumcised, then that will be his choice.”

Some messages may inspire the mobilization of health and social care organizations, thus constituting a call for collective action.

“We particularly have more boys on the waiting lists [for Big Brothers] than we do girls. One of the reasons is because younger men often worry they become a surrogate father when they engage in these mentoring relationships…”

The remaining three messages relate to reorienting health services to better attend to men’s health vulnerabilities, and comments about the political implications in reports of men’s health research.

“But HPV can also threaten the lives and futures of young men… almost 100 per cent of head and neck cancer in men under the age of 40…also been linked to rectal cancer.”

None of the key messages related to building healthy public policy, strengthening community action, or creating supportive environments; this underscores the necessity of a broad political perspective regarding youth’s and men’s health. Summarizing, informative and awareness-raising messages are the newspapers’ predominant discourse on men’s health, mostly addressing prostate and sexual health. News reports were mostly silent about solutions to publicized problems related to political issues, access to health services, and collaborations or inter-sectorial actions. By limiting reporting to the results of research studies and describing initiatives, newspapers barely comment on government actions, or intentions to create a boys’, youth’s and men’s health policy, and did not address issues of citizen participation or engagement. Therefore, the potential for newspapers to influence the mobilization of a collective awareness of the political implications of men’s health issues is unrealized.

### Potential impact of media discourse: General public’s buy-in and support for the development of a national boys’, youth’s and men’s health policy

The dominant messages on men’s health, as shown in Table 3, indicate that research results as publicized by the newspapers still reflect the view point of traditional masculinity in terms of the ideals that a man is expected to achieve and the related risky behaviour. The notion of risky behaviour also poses a social danger for others. Therefore, it is a matter of individual responsibility for men to autonomously change their risky behaviour and personal health. As it seems that little statistic information is available on such risky behaviour, media professionals may lack access to statistical information that reflects the reality. Consequently, the health status analysis, an important preliminary planning step before disseminating evidence-based news is compromised. Due to the easy availability of statistical information about delivery of sexual and reproductive health services, the Canadian journalist mainly focuses on those issues, thus unintentionally (or not?) reinforcing the limited scope of awareness of men’s health.

On the other hand, more recently, news reports on how community services are addressing youth’s and men’s mental health and acknowledging the impacts of cultural and economic struggles suggests a form of community mobilization and change in health practices. Both actions have been widely reported by newspapers, revealing the media’s reliance on the capacity of community members to address their own health and well-being issues, and responding to the influences of gender and collective conceptions of health/illness. In addition, community initiatives have emerged to create social support networks to overcome the effects of men’s abusive behaviour and fragile social bonds. News reports about social learning through community initiatives to help men develop their fathering skills, and to self-manage aggressive behaviours that may counteract the criminalization of youth’s and men’s acts, may help to reshape the general public’s perception regarding the potential for behavioural change.

News introduces a discourse suggesting that traditional, socially expected youth’s and men’s behaviours can be changed. However, changes in the general public’s attitudes toward more progressive views and forms of masculinity in terms of dealing with health issues may be paradoxically hard to take hold due to the multicultural nature of Canadian society. While for the general population, and particularly immigrant men, representations of male identity and social practices related to health matters still embody less attention to health. In this conversation, a combination of individual characteristics and specific SDH such as gender, class, sexuality, personal coping skills and views of masculinity should be incorporated.

Newspapers have the potential to educate the general public about how health can be jeopardized by the attributes of traditional masculinity; they can demonstrate that masculine behaviour is a cultural, rather than a natural, asset—a learned aspect of one’s intergenerational cultural capital, which is at the basis of one’s individual social life while determining one’s position in a given social order. Thus, new learning about health can unfold within a continuum from traditional to a modern masculinity, allowing men to learn about self-care, prevention, medical care seeking skills, disclosure of emotions, responsible fatherhood, and egalitarian relationships that could improve overall quality of life and overall health.

Popular newspapers could also disseminate information to promote political literacy about how community groups are multiplying social advocacy initiatives at several levels. Men’s health interest groups, although small in number in Canada, face a lack of consensus in the political agenda^20^ even though this moment is extremely promising to fuel the discussion about men’s health. If these groups would agree to work together, they could establish research partnerships with scholars to explore the socio-economic dimensions and impacts of boys’, youth’s and men’s health.

## Discussion

The results regarding media content demonstrates that Canadian newspapers focus on health information, awareness, and prevention; together, this accounts for 80% of the analyzed content. The newspaper content also provoked general readers’ thoughts about future action strategies, thus, translating knowledge into pathways for solutions. All of the newspaper articles included in the analysis focused on health (mental/physical health and their social determinants) as related to boys, men and youth, guided by our interest in exploring evidence to address the CIHR call for action. It was identified that these newspaper articles can contribute to readers’ awareness of the health issues faced by the male population, mainly by providing information rather than providing advice or suggesting preventative actions.

Health information presented by the newspapers in a useable format, can allow men with compromised SDH (e.g., education, income and socioeconomic status) to become more conscious health learners, in a manner mirroring Freire’s critical pedagogy for autonomy and critical awareness.^29^ Furthermore, it is foreseeable that, collectively, men can further transform health information into more relatable forms within their peer groups, creating a network of novice knowledge brokers, who simultaneously integrate themselves into the process of translating knowledge while learning new information and teaching skills.

A traditional view of masculinity may be harmful for men’s health seeking behaviour (information and services-related) due to the underlying assumptions that men should take risks and demonstrate strength and invulnerability.^3^ Given such perceptions of masculinity, Canadian newspapers’ discourse about men’s health issues can be understood through the lens of sociocultural communication,^30^ which states that communication produces and reproduces the society’s shared values, beliefs and other culturally-grounded patterns. Of relevance here is the focus on sexual health as the core of men’s health concerns, as identified by the analysis of newspaper article contents. Some newspapers reports did acknowledge the struggles of socially deprived boys and youth, and of men belonging to ethno cultural minorities, thus promoting awareness and consideration of humanistic values. Some attitudes and beliefs were reported that raise doubts about the requisite knowledge, supportive network, resources, capacities and skills, and, ultimately, the self-agency of boys, youth and men to take action to protect and maintain their health.

The newspaper contents used a masculinity-tailored discourse that overlooked the potential to reconceive masculinity as an asset to be mobilized and used as a healthy resource. Dissemination of health information drawn from legitimate resources has the potential to influence men’s behaviour for the better; reliable reporting is important since men have become weary of the poor trustworthiness of some resources. In depth discussion about media and views of masculinity and the effect over public awareness is beyond the scope of this section.

The impact of information in newspapers tied to public awareness could not be measured, assessed or public reactions identified. However, the use of media content analysis in this study illuminated the type of discourse and argumentation used by Canadian newspapers to disseminate research evidences and anecdotal facts that could nourish the public debate about children’s, youth and men’s health and reshape their awareness.

**Figure 1:**
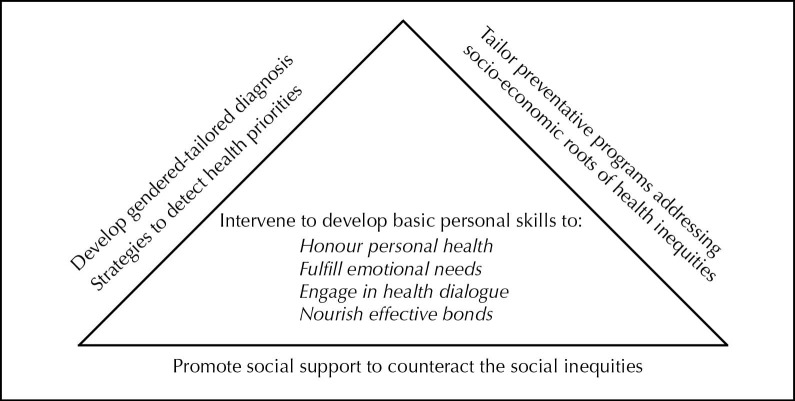
A conceptual framework for comprehensive action-strategies to promote boys’, youth’s and men’s health.

Some indirect impacts are foreseeable on the professional practice of health promotion in concert with different sectors of society requiring concomitant professional initiatives to support the development of personal health skills and reorientation of health services to accommodate and respond to health needs and challenges. For that, we proposed a conceptual framework for integrated, comprehensive action-strategies (see Figure 1) developing thus the male clientele’s personal skills in a way that will achieve four major outcomes: honour personal health, fulfill emotional needs, engage in health dialogue and nourish affective bonds.

## Strengths and limitations

The main strengths relate the generated evidences of the media’s contribution to society’s engagement in the growing debate among researchers about the expansion of research programs on boys’, youth and men’s health, providing thus, support for the development of men’s health policies in Canada. Major limitations are due the fact that the analyzed articles on men’s health were from the most popular English language newspapers distributed in major Canadian urban centres, therefore, results may not be representative of contents from community newspapers that may have more influence on readers living under conditions of greater social vulnerabilities and health inequities. This includes individuals who do not read English or who prefer other types of media such as radio, multicultural television, or digital media.

Another relevant limitation is the incapacity to identify and explore bias related to commercial influence, gender-shaped stereotypes as generated by newspapers’ editors and journalists who produce such messages. Therefore, readers should be cautious when inferring about the actual scope of contribution, impact and influence of newspapers on the overall male population.

Boys, youth (even if they can be less attracted to such type of media) and men can learn about credible sources of health information, and decode health information’s meaning as well as safely incorporate it into their lives, thereby becoming more health literate to effectively deal with risk factors, reduce the incidence and severity of physical and mental diseases, and better self-manage existing health conditions.^11^ The media cannot present policy evidence if it is not provided in research reports, but even in the absence of scientific evidence one can suggest political ideas. Ultimately, the open arena for debate will also educate, engage and mobilize the general public to create change and challenge policymakers.

In sum, we uncovered that newspapers minimally address policy solutions to issues of limited access to services or inter-sectoral collaborations regarding boys’, youth’s and men’s health and social wellness. The scarce political contents in news may reflect no data on government action and a lack of citizen engagement toward the creation of a concerted men’s health policy. By limiting reporting to the results of research studies and describing initiatives, newspapers barely comment on government actions, or intentions to create such policy. Therefore, no instigation to citizen participation or engagement jeopardizes the media influence on the mobilization of a collective awareness of the political implications of men’s health issues.

## Ethical approval

Not applicable. No human subjects participated in this study.

## Competing interests

The authors declare that there is no conflict of interests.

## Disclaimer

The authors claim that no part of this manuscript has been copied from other sources.

## Funding

None.

## Authors’ contributions

MZ, KJ and DS contributed to the design of the work. KJ and DS conducted the data retrieval. AAB contributed to the interpretation of data for the work; and drafting the manuscript. All authors contributed to the analysis and interpretation of data for the manuscript, as well as approved the final version to publish.

## Acknowledgements

The authors would like to acknowledge Andy Bianco, Barrie Zwicker, Dr. Rachelle Miele and Dr. Iraj Poureslami for reviewing an early draft of the manuscript, as well as Dr. Sylvia Novac for editing it.

**Table 1:** Main health topics discussed in retrieved articles by news source, Canada between January 2010 and February 2014

**Main Topic**	**National Post**	**Globe and Mail**	**Metro News**	**Sub-total**
Prostate health & sexual health	5	1	5	11 (25%)
Lifestyle & diet	3	1	4	8 (18%)
Roles & relationships	3	0	5	8 (18%)
Sexual orientation	1	0	4	5 (11%)
Mental health	3	0	2	5 (11%)
Social environment & health	1	0	3	4 (9%)
Seeking medical attention	0	0	3	3 (7%)
Total	16 (36%)	2 (4%)	26 (60%)	44 (100%)

Data were presented using No. (%) for categorical variables.

**Table 2:** Articles’ message goals by news source, Canada, between January 2010 and February 2014

**Message Goal**	**National Post**	**Globe and Mail**	**Metro News**	**Number of entries**	**Percentage**
Provide information	16	2	25	43	37.4%
Provoke awareness	12	2	18	32	27.8%
Elicit prevention	6	1	11	18	15.6%
Unclear messages	3	0	11	14	12.2%
Introduce politics	3	0	5	8	7.0%
Total	40 (36%)	5 (4%)	70 (60%)	115	100%

Data were presented using No (%) for categorical variables. Note: Unclear messages were those whose contents were mixed or undistinguishable.

**Table 3:** Newspaper content related to the social determinants of health

**Core statement**	**Social determinants of health**
- Men avoid seeking medical attention. - Unhealthy diet, lifestyles, and stress can lead to erectile dysfunction, and lowered sperm count as well as the development of abnormal sperm. - Women tend to talk about health issues more than men, though there is a gap in funding and awareness because […] most men are apathetic toward their health and do not want to talk about it. - Men need to be more comfortable seeking help within regards to erectile dysfunctions and sexual health- related issues. - Men carry more bacteria because they may be less disciplined with hand hygiene. - First Nations children use emergency services the most out of all population sub-groups due to seeking mental health care services secondary to substance abuse or self-harm. - Older rappers want to compete with new artists though most in their demographic never thought to live past thirty, and therefore rappers must take appropriate health precautions. - Some Jewish parents believe that circumcision may be seen as abuse or mutilation, and may cause psychological trauma, if it is done without their son’s explicit consent.	Personal health practices (33%)
- Pedophiles are trying to argue that pedophilia is more of a sexual orientation because it is genetically linked. There are support group websites that hope to contact struggling young pedophiles to support them and find ways of controlling problematic behaviour. - There are more boys than girls on the waiting list in the Big Brothers / Big Sisters club, as there is a lack of male volunteers, apparently because young men do not want to feel they are surrogate fathers. - Some humanities and social studies undergraduate students may argue that the ‘Movember’ moustache movement overshadows the actual cause of fundraising for domestic violence services. - “Break the cycle” was created to inform young men of the issue of domestic violence and involve them by challenging any notion that they can do whatever they want to women.	Social support networks (17%)
- HPV immunization with boys will help prevent transmission. - Health Canada has lifted the lifetime ban on gay men donating blood on the condition that the individual hasn’t had sex with another man in 5 years. - The Canadian justice system needs to examine and update their standards and tools in mental health assessment in order to protect and provide the necessary care for individuals that suffer from mental health issues.	Health services (12.5%)
- Women live longer than men due to certain sex hormones that impact the immune system. - Men are just as likely as women to suffer from mental health issues though they may display different symptoms than the traditional ones, which leads to under-diagnosis. - The future of genetic testing and nomograms may improve early detection and reduce the severity of prostate cancer.	Biology and genetics (12.5%)
- Teenage binge drinking has decreased, although not among gay or bisexual boys, possibly due to strong social pressures. - Suicide rates among Inuit boys have risen in the past few years while other males have experienced a decline in suicide-related deaths; initiatives and prevention programs must be properly tailored to this at-risk group to improve their socio-economic health.	Coping skills (8%)
- The effectiveness of the HPV vaccine for boys is in the discussion phase of program implementation and effectiveness in preventing cancer. - The idea of a strong mother-son bond has usually been dismissed because it is deemed weak and feminizing, though studies show this bond is beneficial and makes the sons better communicators.	Healthy child development (8%)
- Although designated high-priority neighbourhoods have received some funding, which did decrease violence, a holistic targeted approach for youths in marginalized neighbourhoods is important.	Physical environment (4%)
- The era of estranged fathers is changing; more fathers are knowledgeable and involved in their children’s lives.	Social status (4%)

Note: SDH classification follows that described by Hamilton and Bhatti.^21^

**Table 4:** Top 10 key media messages about the health of boys and men

**Top key messages**	**Comprehensive action strategies**
- Men avoid talking about their health. - Men tend to be less active regarding health issues. Men avoid seeking medical attention and getting regular check-ups that may prevent premature deaths. - Men’s unhealthy diet and lifestyle can lead to erectile dysfunction; they should visit their doctor to get a full health assessment. - Men involved in domestic violence are being listened to, to understand the roots of their expectations. - Fathers have become more knowledgeable about, and involved in, their children’s lives. - Young men are not sought as volunteer social supports for fatherless boys.	Develop personal skills
- Young men living in low-income, poorly-serviced neighbourhoods have benefited from funding to tackle systemic inequities and reduce violence. - Boys are more likely to be misdiagnosed in relation to eating disorders. - Suicide initiatives and prevention programs must be properly tailored to Inuit boys, who are at high risk.	Reorient health services
